# Numerical investigation of the effect of the opening mode on the pressure relief process of engine nacelle

**DOI:** 10.1038/s41598-022-24419-8

**Published:** 2022-11-30

**Authors:** Yan Yan, Chen Chen, Xiaotian Peng, Chenchen Wang, Shiyu Feng

**Affiliations:** 1 College of Mechanical and Electrical Engineering, Xi’an Polytechnic University, Xi’an, People’s Republic of China; 2 Key Laboratory of Aircraft Environment Control and Life Support of MIIT, College of Aerospace Engineering, Nanjing University of Aeronautics and Astronautics, Nanjing, People’s Republic of China; 3 Jiangsu Key Laboratory of Process Enhancement and New Energy Equipment Technology, School of Mechanical and Power Engineering, Nanjing Tech University, Nanjing, People’s Republic of China; 4 Aviation Key Laboratory of Science and Technology On Aero Electromechanical System Integration, Nanjing Engineering Institute of Aircraft Systems, Aviation Industry Corporation of China, Nanjing, People’s Republic of China

**Keywords:** Aerospace engineering, Mechanical engineering

## Abstract

The pressure relief door (PRD) is a vital structure to ensure the safety and reliability of the engine. This paper established a zero-dimensional transient simulation mathematical model to study the plenum compartment pressure threshold and maximum opening angle effects on the nacelle pressure relief process under different opening modes. Then, a computational fluid dynamics model verified by experimental literature data was used to simulate the nacelle pressure relief process and to determine the influence of two different opening modes on the force and discharge characteristics of the PRD. The results of this study show that different opening modes strongly impact the nacelle pressure relief process. Reducing the nacelle compartment pressure threshold of the PRD opening can reduce the time required for the pressure relief process to reach the equilibrium stage. Reducing the maximum opening angle may increase the nacelle compartment pressure during the equilibrium stage. In addition, under the same nacelle compartment pressure thresholds and maximum opening angles, the pressure relief process under the vertical opening mode can reach a lower nacelle compartment pressure during the pressure relief equilibrium stage compared to that under the horizontal opening mode. Therefore, the vertical opening mode is better than the horizontal opening mode. This paper provides two lower calculation costs and high accuracy research models for studying the nacelle pressure relief process.

## Introduction

The nacelle of an aircraft contains engine accessories, such as the engine, casing, booster pump, and various sensors. The pressure change in the engine nacelle will significantly impact the working conditions of susceptible electronic components, fuel lines, circuits, etc. During the flight of an aircraft, if the engine's air intake pipe is damaged so that a large amount of high temperature and high-pressure gas leaks and the leaked gas cannot be discharged from the nacelle in time, the internal pressure of the nacelle will rapidly increase. Excessive force can damage the nacelle structure and even the engine. According to the US Federal Aviation Regulation (FAR) Part 25.1103 (d)^1^ provision, “For turbine engine and auxiliary power unit bleed air duct systems, no hazard may result if a duct failure occurs at any point between the air duct source and the airplane unit served by the air”. Therefore, a pressure relief door (PRD) must be installed on a nacelle that opens after the internal pressure of the nacelle increases to a certain threshold, which will maintain the internal pressure in the core casing of a high-bypass turbofan engine under a safe level to avoid structural damage or failure of the nacelle structure. In recent years, civil aviation engines have developed in the direction of a high bypass ratio and overall pressure ratio, and the fan pressure ratio has gradually decreased^2,3^. Compared with aircraft from the 1950s, the operating pressure and temperature of modern aircraft engines are higher, which has led to a significant increase in emissions from the nacelle pressure relief system: after the engine bleed air duct is ruptured, the nacelle structure will bear a higher pressure load, which in turn, will result in higher requirements for the discharge performance of the PRD^4,5^. Therefore, it is essential to design a reasonable structure for a pressure relief valve and understand its operating performance and control method.

The pressure relief characteristics of a nacelle PRD should be understood to determine the maximum pressure in the nacelle of an engine following high-pressure gas leaks. During the operation of an aircraft nacelle, the outflow Mach number and pressure ratio affect the pressure difference. The discharge characteristics between the PRD and the plenum, the shape, opening method, opening angle, and aspect ratio of a PRD influence the gas flow state and force characteristics of the PRD during the nacelle pressure relief process. Therefore, the influence of the above factors needs to be clarified when studying the pressure relief characteristics of the PRD.

In recent decades, there have been few public research reports of experimental and numerical simulations of the pressure relief process of a nacelle. Dewey et al.^6,7^ experimentally measured the discharge characteristics of an auxiliary exhaust port with a particular inclination. The results show that when the discharge flow rate is low, the inclined outlet has a higher emission coefficient than the outlet perpendicular to the airflow. In addition, when the discharge flow coefficient is constant, the outflow Mach number has little effect on the flow coefficient of an outlet with a sloped or curved pipe. Vick et al.^8^conducted an experimental study on the discharge and force characteristics of the auxiliary air outlet of a curved duct with flap discharge in transonic airflow. However, Vick's work mainly focused on the additional ventilation flow, so it was limited to pressure ratios below 1. Pratt et al.^9,10^, and Yu et al.^11^ used the CFD method to numerically study the discharge and force characteristics of a PRD during pressure release from a nacelle and studied the nacelle pressure relief process under different Mach numbers, pressure ratios, and opening angles. This research found that there is an optimal opening angle value and that the discharge coefficient for the PRD reaches a maximum under this opening angle value. Benard et al.^12^ experimentally investigated the pressure relief process for a PRD with a pressure ratio greater than 1. The results showed that the discharge flow ratio decreases with an increasing Mach number at a given pressure ratio. Vedeshkin et al.^13^ performed an experimental study of the discharge and force characteristics of a nacelle PRD and compared the experimental results with numerical simulation results. The results showed a good agreement between the numerical simulation results and the experiment. However, the PRD opening mode in this study is unlike that found in previous research, in which the PRD hinge is oriented parallel to the freestream direction. Schott et al.^14^ studied the effects of the PRD aspect ratio and conducted comprehensive numerical simulations with a series of pressure ratios, Mach numbers, internal temperatures, and other conditions. The results show that the angle of the equilibrium torque increases with increasing pressure in the high-pressure chamber and decreases with an increasing Mach number. The discharge coefficient rises with a rising opening angle and does not increase after reaching a maximum value. In addition, many engineers have proposed various structures for a nacelle PRD and given some suggestions for reference^15–20^.

The nacelle pressure relief process is well known to involve complex transient flow behavior. However, the pressure relief door in many of the above studies is stationary, which cannot reflect the relationship between the plenum compartment pressure and the opening angle of the PRD overtime during the actual pressure relief process^21,22^. Therefore, a new calculation method is proposed in this paper. First, steady-state CFD analysis is used at a fixed opening angle to obtain the relationship between the discharge coefficient *C*_D_ and the moment M under the plenum compartment pressure at a certain freestream Mach number and aspect ratio. Then, the opening angle is changed to obtain the relationship between the discharge coefficient *C*_D_ and the moment M with the opening angle of the PRD. On this basis, a zero-dimensional transient simulation mathematical model of the nacelle pressure relief process is established. The *C*_D_ and M values obtained are substituted into the model for calculation. This method dramatically reduces the calculation cost while ensuring accuracy, so one can better meet actual engineering needs. In addition, there is still a lack of relatively simple simulation models for practical engineering application prediction.

**Figure 1:**
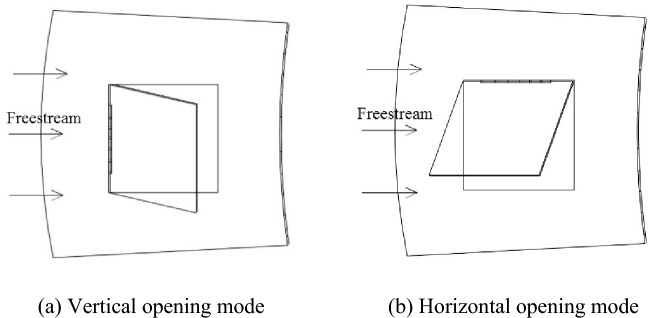
Two opening modes of a nacelle PRD.

Most existing studies are based on the PRD opening mode being a vertical opening mode in which the PRD hinge is oriented perpendicular to the freestream direction, as shown in Fig. 1a. However, few studies are based on a nacelle pressure relief process under a horizontal opening mode in which the PRD hinge is oriented parallel to the freestream direction, as shown in Fig. 1b. Moreover, because the structure of the PRD and the working conditions of the pressure relief process are different, it is difficult to compare the effects of the two different opening modes on the nacelle PRD pressure relief process. Therefore, based on the transient simulation model of the nacelle pressure relief process established above, this paper conducts a detailed calculation and analysis of the effects of the two different opening modes on the nacelle PRD pressure relief process for the same PRD structure and working conditions to understand the difference in the pressure relief performance of the PRD under the two different opening modes.

## Mathematical model of the nacelle pressure relief process

### Simplification and assumptions

**Figure 2:**
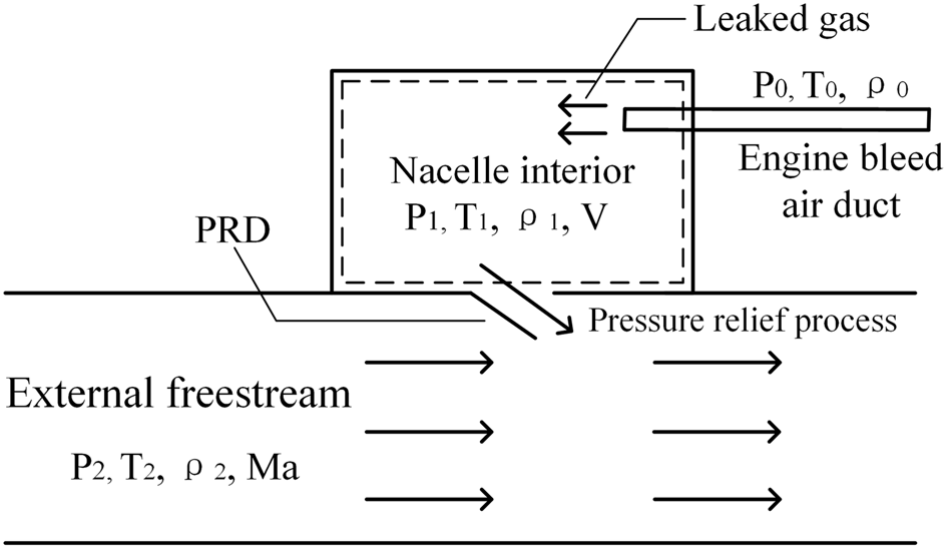
Schematic diagram of the pressure relief process.

When the engine bleed air duct is broken and has a severe leak, the leaked gas will flow into the nacelle, increasing the internal pressure of the nacelle above the threshold. Then, the PRD will open, and the pressure relief process will occur under the collective effect of the plenum compartment discharge gas and external free stream. In addition, the pressure relief process will also be affected by factors such as the PRD opening mode, aspect ratio, and opening angle. Therefore, the pressure relief process is a complex flow process with a multi-factor coupling, and the flow diagram of the pressure relief process is shown in Fig. 2. To simplify the simulation, some unimportant factors are ignored, and one makes the following assumptions.

1. The pressure in the high-pressure bleed air duct and the external ambient pressure and temperature of the nacelle remain unchanged during the entire pressure relief process.
2. Air is considered an ideal gas, and heat transfer is not considered during the pressure relief process. Therefore, it is an adiabatic process.
3. It is considered that the thermal parameters, such as temperature, pressure, and density, in the control volume of the nacelle are uniform.
4. The internal gas discharge during the pressure relief process is discharged through the PRD, i.e., there are no other gas leaks.
5. The PRD is sufficiently rigid, so there is no need to consider the effect of its deformation on the pressure relief process.

### Zero-dimensional transient simulation mathematical model

When *p*_1_/*p*_0_ > *π*_p,cr_, the flow is in a noncritical state, and the leakage mass flow rate for the high-pressure bleed air duct is given by

1 $$ q_{{{\text{m}},0 - 1}} = \mu A_{0} \left\{ {\frac{2\gamma }{{\gamma - 1}} \cdot \frac{{p_{0}^{2} }}{{RT_{0} }}\left[ {\left( {\frac{{p_{1} }}{{p_{0} }}} \right)^{{\frac{2}{\gamma }}} - \left( {\frac{{p_{1} }}{{p_{0} }}} \right)^{{\frac{\gamma + 1}{\gamma }}} } \right]} \right\}^{\frac{1}{2}} $$

When *p*_1_/*p*_0_ ≤ *π*_p,cr_, the flow reaches a critical state, and the leakage mass flow rate for the high-pressure bleed air duct is given by

2 $$ q_{{{\text{m}},0 - 1}} = \mu A_{0} \left\{ {\left( {\frac{2}{\gamma + 1}} \right)^{{\frac{\gamma + 1}{{\gamma - 1}}}} \left( {\frac{{\gamma p_{0}^{2} }}{{RT_{0} }}} \right)} \right\}^{\frac{1}{2}} $$

where *μ* is the flow coefficient of the high-pressure bleed air duct leakage. *A*_0_ is the leakage area. *R* is the specific gas constant (for air, R = 287.1 J/(kg·K)). *p*_0_ is the pressure in the high-pressure bleed air duct. *T*_0_ is the temperature of the high-pressure bleed air duct. And *p*_1_ is the nacelle interior pressure.

When *p*_2_/*p*_1_ > *π*_p,cr_, the flow is in a noncritical state, and the discharge mass flow rate for the PRD is given by

3 $$ q_{{{\text{m}},1 - 2}} = C_{{\text{D}}} A_{{{\text{door}}}} \sin \varphi \left\{ {\frac{2\gamma }{{\gamma - 1}} \cdot \frac{{p_{1}^{2} }}{{RT_{1} }}\left[ {\left( {\frac{{p_{2} }}{{p_{1} }}} \right)^{{\frac{2}{\gamma }}} - \left( {\frac{{p_{2} }}{{p_{1} }}} \right)^{{\frac{\gamma + 1}{\gamma }}} } \right]} \right\}^{\frac{1}{2}} $$

When *p*_2_/*p*_1_ ≤ *π*_p,cr_, the flow reaches a critical state, and the discharge mass flow rate for the PRD is given by

4 $$ q_{{{\text{m}},1 - 2}} = C_{{\text{D}}} A_{{{\text{door}}}} \sin \varphi \left[ {\left( {\frac{2}{\gamma + 1}} \right)^{{\frac{\gamma + 1}{{\gamma - 1}}}} \left( {\frac{{\gamma p_{1}^{2} }}{{RT_{1} }}} \right)} \right]^{\frac{1}{2}} $$

where *C*_D_ is the discharge coefficient of the PRD under different plenum compartment pressures and opening angles, which is obtained from CFD steady-state simulation calculation. *A*_door_ is the area of the PRD. *φ* is the opening angle of the PRD. *T*_1_ is the internal nacelle temperature. And *p*_2_ is the freestream static pressure of the external environment.

The mass of the gas inside the nacelle varies over time as follows:

5 $$ \frac{{{\text{d}}m_{1} }}{{{\text{d}}t}} = \frac{{{\text{d}}m_{{1,{\text{in}}}} }}{{{\text{d}}t}} - \frac{{{\text{d}}m_{{1,{\text{out}}}} }}{{{\text{d}}t}} = q_{{{\text{m}},0 - 1}} - q_{{{\text{m}},1 - 2}} $$

where *m*_1_ is the gas mass inside the nacelle. *m*_1, in_ is the gas mass flowing in the nacelle. And *m*_1, out_ is the gas mass flowing out of the nacelle.

From the simultaneous adiabatic flow equation *p*/*ρ*^*γ*^ = *C* and ideal gas equation *pV* = *mRT*, the derivative of time can obtain

6 $$ \frac{{{\text{d}}p_{1} }}{dt} = \frac{{\gamma RT_{1} }}{V} \cdot \frac{{{\text{d}}m_{1} }}{{{\text{d}}t}} $$

where *V* is the internal volume of the nacelle.

The nacelle's internal temperature varies over time as

7 $$ \frac{{{\text{d}}T_{1} }}{{{\text{d}}t}}{ = }\frac{{T_{1} }}{{m_{1} }}(\gamma - 1) \cdot \frac{{{\text{d}}m_{1} }}{{{\text{d}}t}} $$

When the PRD rotates, the rotation angle acceleration *α* is

8 $$ \alpha = \frac{{{\text{d}}\omega }}{{{\text{d}}t}}{ = }\frac{{{\text{d}}^{2} \varphi }}{{{\text{d}}t^{2} }} = \frac{{M - M_{f} }}{J} $$

where *ω* is the rotational angular velocity. *M* is the total moment of the PRD, which is obtained from CFD steady-state simulation calculation. *J* is the moment of inertia of the PRD rotating around the hinge, and *M*_*f*_ is the resistance moment when the PRD is turned, i.e.,

9 $$ M_{f} = \frac{1}{8}c\rho_{1} \omega^{2} l^{4} b $$

where *c* is the rotation resistance coefficient of the PRD. *ρ*_1_ is the density of the discharge gas. *l* is the PRD chord length. And *b* is the PRD width.

### Results and discussion

#### Effect of the nacelle compartment pressure threshold on the pressure relief process

**Figure 3:**
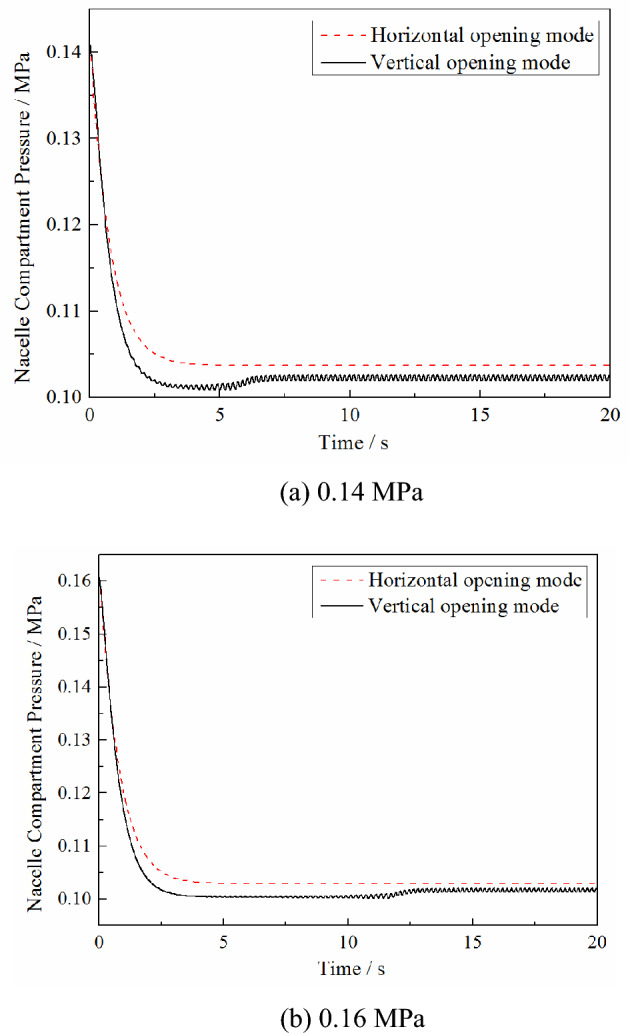
Effect of different opening modes on the nacelle compartment pressure changes under different opening thresholds.

**Figure 4:**
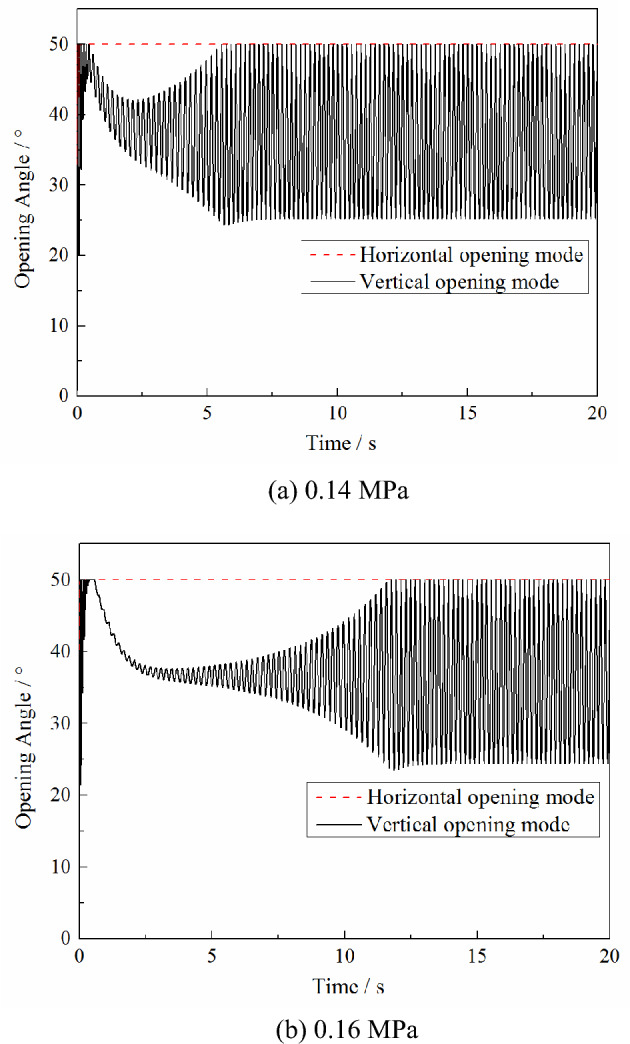
Effects of different opening modes on the changes of the opening angle under different opening thresholds.

The calculation conditions are based on a Mach number of 0.7, pressure in the high-pressure bleed air duct of 0.4 MPa, initial pressure in the nacelle of 0.1 MPa, PRD area of 0.0625 m^2^ and aspect ratio of 1. The maximum opening angle of the PRD is set to 50°, and the nacelle compartment pressure threshold of the PRD opening is 0.16 MPa and 0.14 MPa. The calculated variations in the plenum compartment pressure and PRD opening angle with time under the different opening modes are shown in the Figs. 3 and 4.

As seen in Fig. 3, the pressure relief process under different opening modes is quite different. In the vertical opening mode, due to the PRD being affected by the discharge gas from the nacelle and the external free stream at the same time, a rapid reciprocating swing occurs. However, in the horizontal opening mode, because the force acting on the PRD is entirely due to the discharge gas from the nacelle, the PRD is fixed at the maximum opening angle. As a result, the changes in the nacelle compartment pressure under different opening modes are also various. Compared with the horizontal opening method, the nacelle compartment pressure in the pressure relief equilibrium stage is lower under the longitudinal opening method because the discharge coefficient *C*_D_ of the PRD under the vertical opening mode is higher than that under the horizontal opening mode. However, due to the reciprocating swing in the vertical opening mode, the nacelle compartment pressure will slightly fluctuate during the pressure relief equilibrium stage. In the horizontal opening mode, the nacelle compartment pressure is stable at a specific value because the PRD is stabilized at the maximum opening angle.

In addition, as can be seen from Fig. 4, reducing the nacelle compartment pressure threshold of the PRD opening will reduce the time required for the pressure relief process to reach the equilibrium stage. But it does not affect the changes in the nacelle compartment pressure and the opening angle at the equilibrium stage.

#### Effect of the maximum opening angle on the pressure relief process

**Figure 5:**
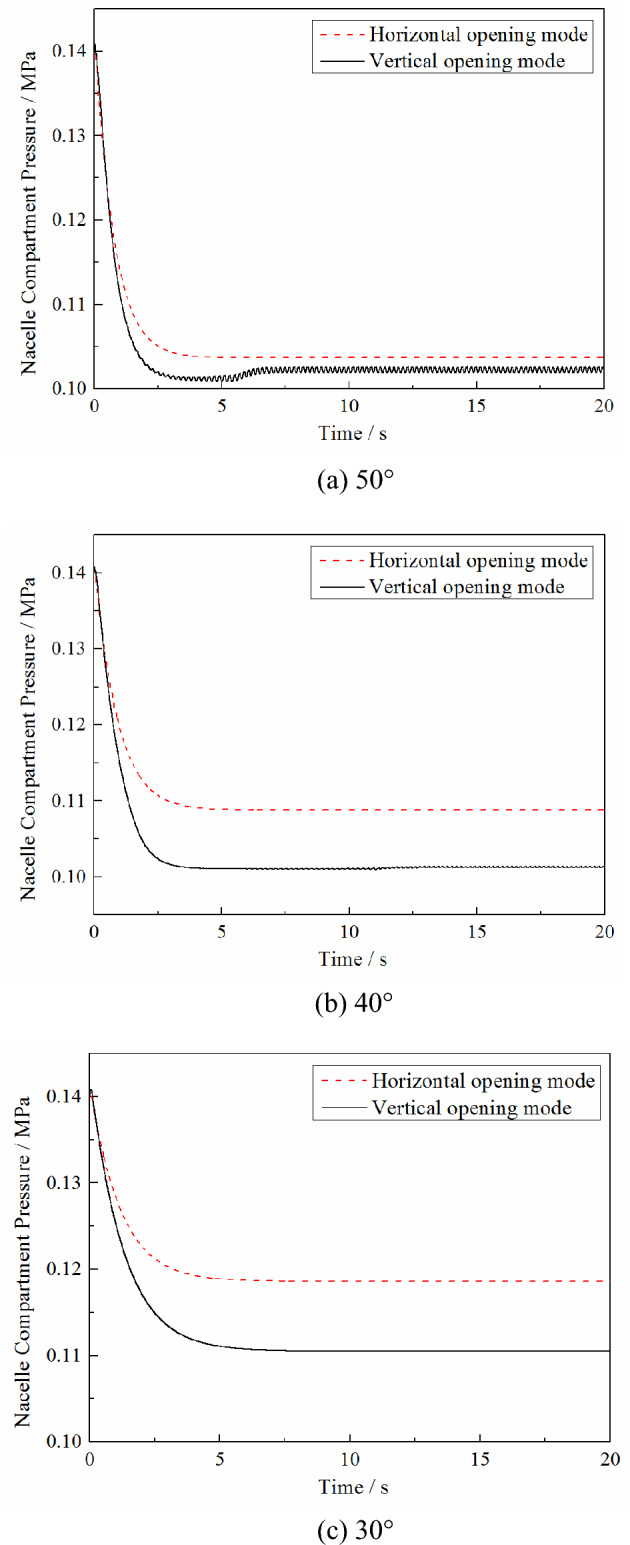
Effects of different maximum opening angles on the changes of the nacelle compartment pressure under different opening thresholds.

**Figure 6:**
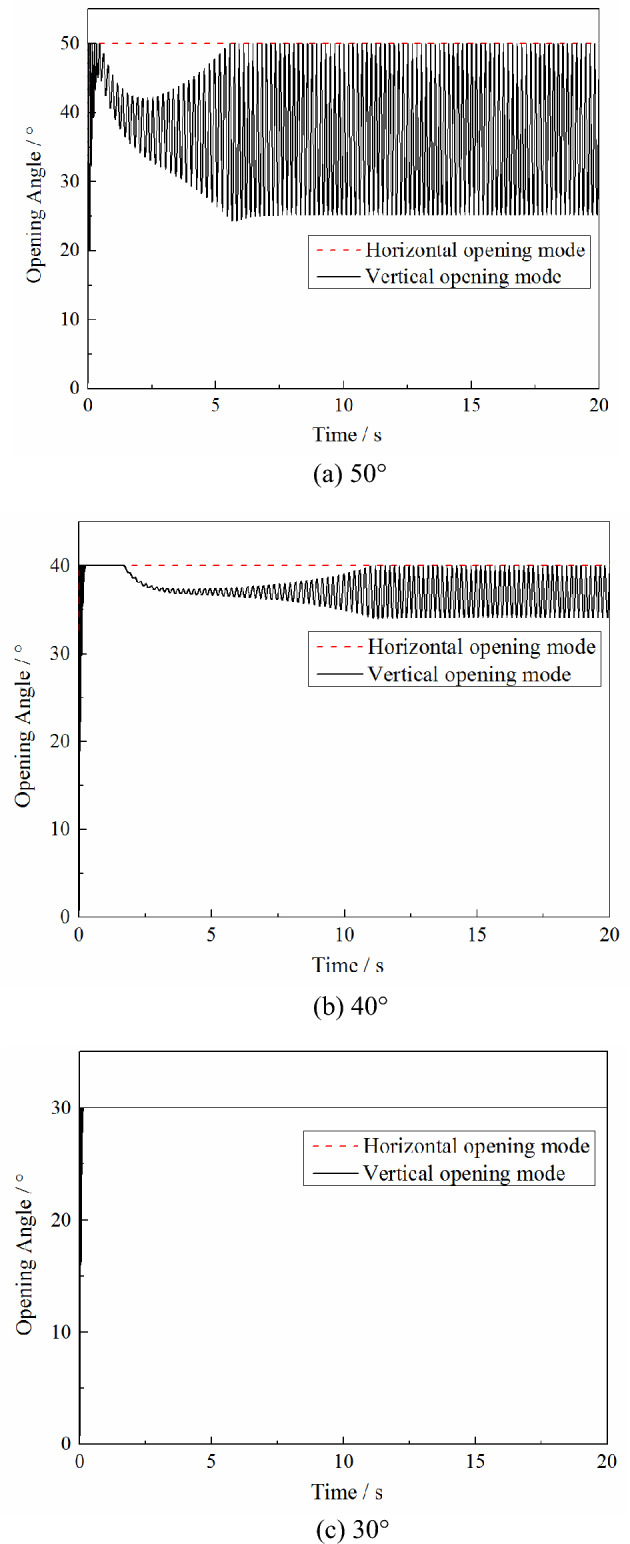
Effects of different maximum opening angles on the changes of the opening angle under different opening thresholds.

The calculation conditions are based on a Mach number of 0.7, pressure in the high-pressure bleed air duct of 0.4 MPa, initial pressure in the nacelle of 0.1 MPa, PRD area of 0.0625 m^2^_,_ and aspect ratio of 1. The nacelle compartment pressure threshold of the PRD opening was set to 0.14 MPa, and the maximum opening angle of the PRD was 50°, 40° or 30°. The calculated variations of the plenum compartment pressure and the opening angle of the PRD with time under the different opening modes are shown in the Figs. 5 and 6.

As seen from Figs. 5 and 6, under different maximum opening angles, compared with the horizontal opening mode, the vertical opening mode can achieve a lower nacelle compartment pressure during the pressure relief equilibrium stage. In the horizontal opening mode, the nacelle compartment pressure at the equilibrium stage increases as the maximum opening angle is reduced. However, in the vertical opening mode, when the maximum opening angle is reduced from 50° to 40°, there is almost no effect on the pressure relief equilibrium stage. And as the maximum opening angle decreases, the nacelle compartment pressure at the equilibrium stage increases.

In the horizontal opening mode, since the moment acting on the PRD is always positive, no matter what the maximum opening angle is set to, the PRD will be fixed at the maximum opening angle during the pressure relief equilibrium stage. However, in the vertical opening mode, as the maximum opening angle decreases, the reciprocating swing angle of the PRD during the pressure relief equilibrium stage decreases.

## Numerical procedure

### Physical model

**Figure 7:**
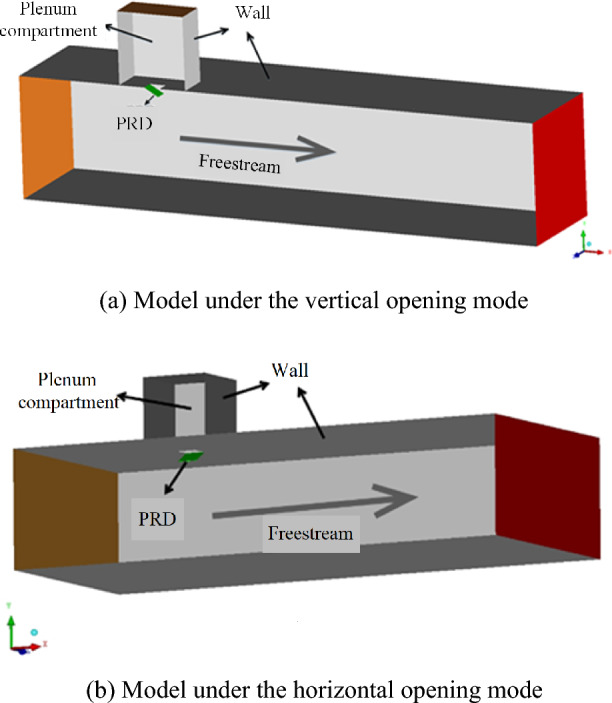
Physical model.

The physical model established in this paper is shown in Fig. 7, in which Fig. 7a shows the vertical opening mode and Fig. 7b shows the horizontal opening mode. The computational domain is a 280 cm × 150 cm × 665 cm cuboid area that is used to simulate the external freestream flow during an airplane flight. The upper part is a plenum compartment with dimensions of 85 cm × 85 cm × 95 cm), simulating a high-pressure area after the bleed air duct is broken. There is a 3 mm gap between the freestream domain and the plenum compartment, which is used to simulate the wall thickness of the PRD.

**Figure 8:**
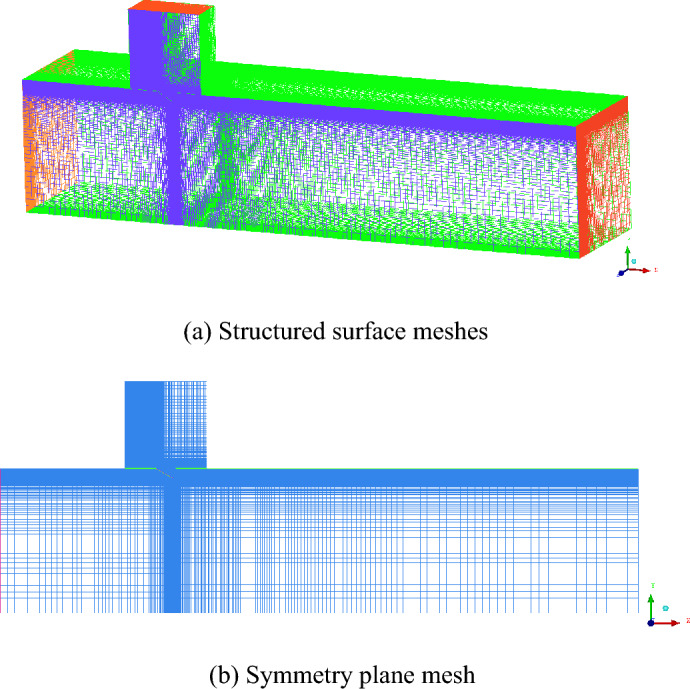
Meshes used for the calculation model.

Since the computational physical model is a symmetrical structure for the vertical opening mode, the computational domain is symmetric. Only one-half was computed, significantly reducing the computational cost while satisfying the calculation. A high-quality structured mesh was obtained for the calculation by ICEM CFD due to the consideration that the boundary layer mesh of the PRD surface needs to be encrypted and that the structure of the flow outlet region is complicated, with the overall mesh shown in Fig. 8. The y + values of the cell centers adjacent to the wall were kept between 30 and 60 under different operating conditions. Mesh numbers of 1.1 × 10^7^, 5.0 × 10^7^, and 14.3 × 10^7^ were calculated under the vertical opening mode to validate the independence of the mesh, and the calculation results are shown in Table 1. The calculation results show that for a mesh number of 5.0 × 10^7^, the error in the simulation results compared with a larger mesh number is minimal. Therefore, the mesh number and the simulation results can be considered to have reached independence.

**Table 1:** Simulation results error under different numbers of mesh.

Mesh number	Discharge coefficient *C*_D_
1.1 × 10^7^	1.058
5.0 × 10^7^	1.029
14.3 × 10^7^	1.032
Error of 1.1 × 10^7^ and 14.3 × 10^7^	2.5%
Error of 5.0 × 10^7^ and 14.3 × 10^7^	0.4%

### Computational method

A computational fluid dynamics calculation is carried out based on a PRD area of 0.0625 m^2^ and an aspect ratio of 1 to obtain the relationship between the discharge coefficient *C*_D_ and the moment M with the opening angle of the PRD. The PRD aspect ratio is defined as the ratio of the door span to the door chord length. There are two ways to open the PRD: the vertical opening mode and the horizontal opening mode. The external freestream Mach number is 0.7, and the plenum compartment pressures *p*_1_ are 0.1 MPa, 0.12 MPa, 0.14 MPa, and 0.16 MPa. Then, cases for PRD opening angles of 10°, 20°, 30°, 40°, and 50° are numerically calculated.

A pressure-based coupled solver was used to solve the compressible Reynolds-averaged Navier–Stokes equations (RANS)^23^, and a second-order upwind discrete scheme was adopted. The realizable k-ε turbulence model was used because it has a known accuracy when dealing with flows involving jets, separations, and secondary flows. Standard wall functions were used to simulate the influence of the wall, and solid walls were assumed to have no speed slip and mass penetration^24^. The commercial ANSYS software package Fluent 19.2 was to solve the calculations.

The plenum compartment and the freestream flow had a uniform total temperature and total pressure distribution. It was assumed that the plenum compartment flow remained static, so the total pressure and static pressure were equal, similar to that found for the high-pressure region after a burst duct event. The outflow boundary condition was set as a uniform static pressure. The freestream's static pressure at the basin's inlet was adjusted to provide the required Mach number, and the total pressure of the plenum compartment was adjusted to study the force and discharge characteristics under different plenum compartments pressures.

### Discharge coefficient formula

Discharge coefficient *C*_D_: The discharge coefficient was calculated based on isentropic relationships assuming an ideal gas. This is an important parameter used to measure the effectiveness of the PRD discharge characteristic at a particular opening angle.

The pressure ratio was calculated using

10 $$ \pi_{{\text{p}}} = \frac{{p_{1} }}{{p_{2} }} $$

where *p*_1_ is the plenum compartment pressure, and *p*_2_ is the freestream static pressure.

The critical pressure ratio is calculated using

11 $$ \pi_{{{\text{p}},{\text{cr}}}} = \left( {\frac{2}{\gamma + 1}} \right)^{{\frac{\gamma }{\gamma - 1}}} $$

where *γ* is the ratio of the specific heat. For air, *γ* = 1.4.

For a pressure ratio *π*_p_ > *π*_p,cr_, the discharge coefficient is calculated using

12 $$ C_{{\text{D}}} = \frac{m}{{A_{{{\text{door}}}} \sin \varphi \left\{ {\frac{2\gamma }{{\gamma - 1}}\frac{{p_{1}^{2} }}{{RT_{1} }}\left[ {\left( {\pi_{p} } \right)^{{\frac{2}{\gamma }}} - \left( {\pi_{p} } \right)^{{\frac{\gamma + 1}{\gamma }}} } \right]} \right\}^{\frac{1}{2}} }} $$

When the pressure ratio *π*_p_ ≤ *π*_p,cr_, the discharge coefficient is calculated as:

13 $$ C_{{\text{D}}} = \frac{m}{{A_{{{\text{door}}}} \sin \varphi \left[ {\left( {\frac{2}{\gamma + 1}} \right)^{{\frac{\gamma + 1}{{\gamma - 1}}}} \left( {\frac{{\gamma p_{1}^{2} }}{{RT_{1} }}} \right)} \right]^{\frac{1}{2}} }} $$

where *m* is the actual mass flow rate through the outlet, *A*_door_ is the area of the PRD, *φ* is the opening angle of the PRD, *R* is the specific gas constant (for air, *R* = 287.1 J/(kg·K)), and *T*_1_ is the plenum compartment temperature.

### Model validation

**Figure 9:**
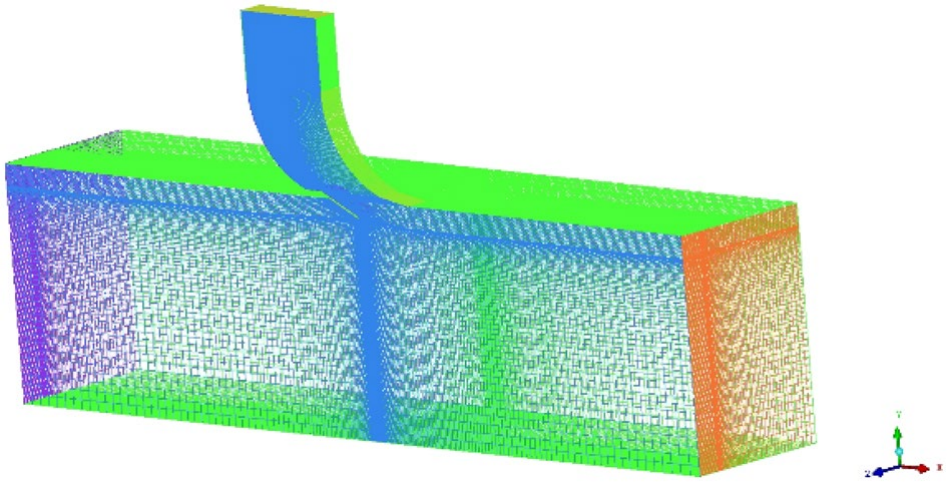
Structured mesh for the experimental equipment.

**Figure 10:**
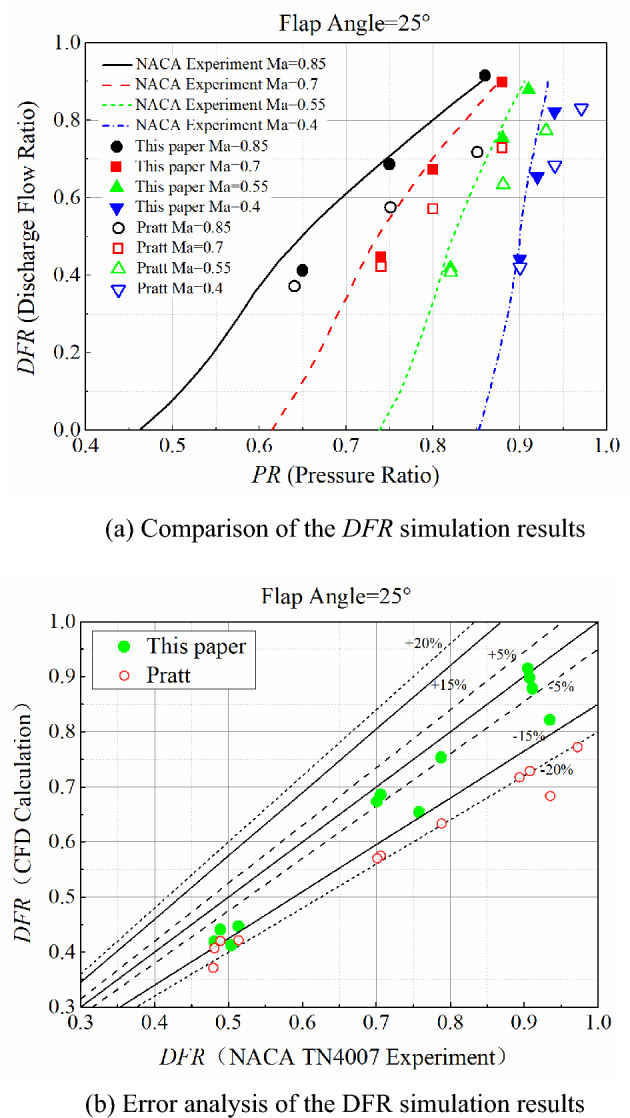
Comparison and error analysis of the simulation results and experimental data for DFR.

The existing experimental data were compared with the numerical simulation results obtained in this paper to verify the accuracy and correctness of the mathematical models and numerical methods. The experimental work described in NACA TN4007^8^ was obtained by numerical simulation using the above method. The mesh is shown in Fig. 9, and the discharge flow ratio (*DFR*) was extracted from the numerical calculations for a single angle and plotted in Fig. 10a,b, respectively, which includes the corresponding data from NACA TN4007 and the simulation results of Pratt et al.^10^. *DFR* is defined as:

14 $$ DFR = \frac{m}{{\rho {}_{2}U_{2} A}} $$

where *m* is the mass flow rate through the plenum compartment outlet, *U*_2_ is the freestream velocity, and *ρ*_2_ is the freestream density. A is the effective outlet area, defined as the minimum cross-sectional area between the outlet wall and the door.

The pressure ratio (*PR*) is defined as:

15 $$ PR = \frac{{p_{1} }}{{p_{2,t} }} $$

where *p*_2, t_ is the freestream total pressure. The results show that the numerical simulation results match the curve trend observed from the experimental data. Furthermore, the simulation results obtained in this paper are closer to the experimental data than those of Pratt et al.^10^. The simulation results have an error of less than 4.9% and a maximum error of no more than 19.8%, which indicates the established model has high accuracy. The total average error is about 10%.

### CFD simulation results

**Figure 11:**
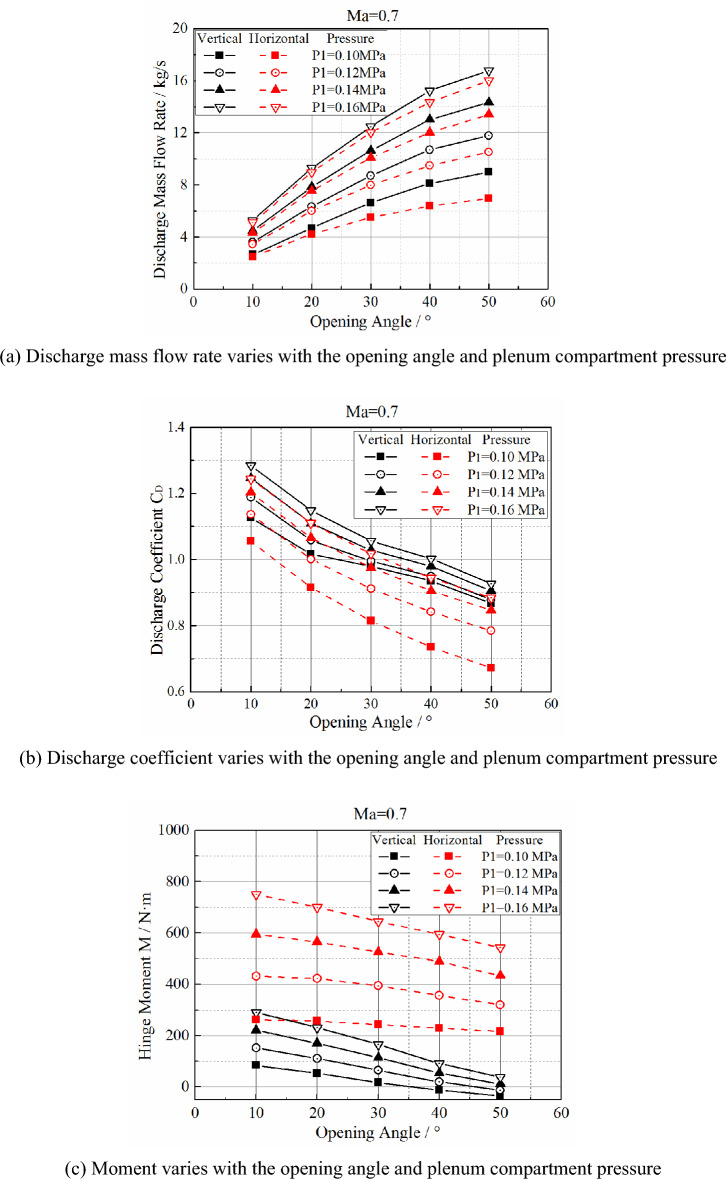
Effects of opening angle and plenum compartment pressure on PRD discharge performance.

Under different plenum compartment pressures and opening angles, the simulation results obtained for the relationship among the PRD discharge mass flow rate, discharge coefficient *C*_D,_ and hinge moment *M* with the PRD opening angle and plenum compartment pressure are shown in Fig. 11.

It can be seen from the Fig. 11a,b that under different opening modes and plenum compartment pressures, the nacelle PRD discharge mass flow rate increases as the opening angle increases, and the changing rate decreases with an increasing opening angle. The discharge coefficient *C*_D_ shows a downward trend with the opening angle increases. At different opening angles and plenum compartment pressures, the PRD discharge mass flow rate and the discharge coefficient *C*_D_ in the pressure relief process under the vertical opening mode are higher than those under the horizontal opening mode.

As shown in Fig. 11c, under the different opening modes, the moment M of the nacelle PRD decreases with an increasing opening angle and increases with a rising plenum compartment pressure. Under the vertical opening mode, there is an equilibrium angle since the force acting on the PRD from the plenum compartment discharge gas and the external freestream is opposite and counteracts a part of the torque under its combined effect. At this opening angle, the moment acting on the PRD is zero, and the equilibrium angle value increases as the plenum compartment pressure increases. However, a different situation is found in the horizontal opening mode because the moment acting on the PRD arises from the plenum compartment discharge gas. Therefore, there is no equilibrium state at a certain angle similar to the vertical opening mode. That is, the moment acting on the PRD will not be reduced to zero, and no negative value will appear. In addition, under different opening angles, the moment operating on the PRD under the horizontal opening mode is higher than that in the vertical opening mode, which is approximately two to three times that in the vertical opening mode.

## Conclusions

1. Different opening modes will significantly impact the discharge and force characteristics of a nacelle PRD. The discharge coefficient *C*_D_ of a PRD under the vertical opening mode is higher than that under the horizontal opening mode. The pressure relief process reaches a lower nacelle compartment pressure during the pressure relief equilibrium stage under the vertical opening mode compared with that under the horizontal opening mode. In conclusion, the vertical opening mode is better than the horizontal opening mode.
2. Reducing the nacelle compartment pressure threshold of a PRD opening does not affect the nacelle compartment pressure and opening angle changes at the equilibrium stage. Still, it can reduce the time required for the pressure relief process to reach equilibrium. So decreasing the nacelle compartment pressure threshold is recommended.
3. In the vertical opening mode, appropriately reducing the maximum opening angle can effectively mitigate the PRD reciprocating swing angle in the equilibrium stage and does not affect the nacelle compartment pressure in the equilibrium stage. Therefore, the pressure relief efficiency can be improved by appropriately reducing the maximum opening angle.

However, the mathematical model established in this study is relatively limited. For example, the numerical model used is made of simple geometric constructions, which is different from the complex shape of the actual engine compartment. Therefore, it is necessary to conduct a more detailed model to make it more practical and explore the stress change and pressure relief flow mechanism of the PRD in future work.

## Data Availability

Some or all data, models, or codes that support the findings of this study are available from the corresponding author upon reasonable request.
