# Author Correction: Numerical investigation of the effect of the opening mode on the pressure relief process of engine nacelle

**DOI:** 10.1038/s41598-023-38931-y

**Published:** 2023-07-26

**Authors:** Yan Yan, Chen Chen, Xiaotian Peng, Chenchen Wang, Shiyu Feng

**Affiliations:** 1 College of Mechanical and Electrical Engineering, Xi’an Polytechnic University, Xi’an, People’s Republic of China; 2 Key Laboratory of Aircraft Environment Control and Life Support of MIIT, College of Aerospace Engineering, Nanjing University of Aeronautics and Astronautics, Nanjing, People’s Republic of China; 3 Jiangsu Key Laboratory of Process Enhancement and New Energy Equipment Technology, School of Mechanical and Power Engineering, Nanjing Tech University, Nanjing, People’s Republic of China; 4 Aviation Key Laboratory of Science and Technology On Aero Electromechanical System Integration, Nanjing Engineering Institute of Aircraft Systems, Aviation Industry Corporation of China, Nanjing, People’s Republic of China

Correction to: *Scientific Reports* 10.1038/s41598-022-24419-8, published online 30 November 2022

The Funding section in the original version of this Article was omitted. The Funding section now reads:

“This study was made possible by Natural Science Basic Research Program of Shaanxi (ProgramNo. 2023-JC-QN-0154)”

The original Article has been corrected.

